# Daprodustat: A potential game-changer in renal anemia therapy—A perspective

**DOI:** 10.3389/fphar.2023.1249492

**Published:** 2023-08-10

**Authors:** Muhammad Usman Haider, Muhammad Furqan, Qasim Mehmood

**Affiliations:** Department of Medicine, King Edward Medical University, Lahore, Punjab, Pakistan

**Keywords:** anemia, chronic kidney disease, daprodustat, HIF, renal disease

## Abstract

The United States FDA has approved daprodustat (DPD) as the first oral treatment option for anemia due to chronic kidney disease (CKD) in dialysis patients. Clinical trials have demonstrated DPD’s efficacy and safety, showing non-inferiority to darbepoetin and suggesting reduced IV iron usage. DPD also holds potential for treating chronic kidney disease anemia in non-dialysis patients and may have benefits for patients with coexisting renal anemia and heart failure, pending further research and trials.

## Introduction

The United States Food and Drug Administration (FDA) has approved daprodustat (DPD) for treating anemia due to chronic kidney disease (CKD) for patients undergoing dialysis for at least 4 months (FDA Approves First Oral Treatment for Anemia Caused by Chronic Kidney Disease for Adults on Dialysis | FDA, 2023). This is a landmark step in managing anaemia due to CKD, as it is the first and only approved oral treatment option. Oxygen-dependent inhibition of HIF-prolyl hydroxylase enzymes by these molecules leads to the buildup of HIF-α, which in turn increases the expression of genes responsive to HIF, such as EPO and those related to iron metabolism and utilization. It would increase the endogenous production of erythropoietin, decreases hepcidin levels in circulation, and ultimately promotes higher levels of red blood cell production and increased hemoglobin concentrations in patients with End Stage Renal disease. (Akizawa et al., 2017). The ASCEND-D (Anemia Studies in CKD: Erythropoiesis via a Novel PHI DPD-Dialysis) Trial is the basis for this approval (Anemia Studies in Chronic Kidney Disease: Erythropoiesis Via a Novel Prolyl Hydroxylase Inhibitor Daprodustat-Dialysis ASCEND-D - Full Text View - ClinicalTrials.gov, 2023). The ASCEND Phase 3 clinical program, which continued for 4 years, assessed the safety and effectiveness of DPD, and its results were published in December 2021 in The new england jounal of medicine (Singh et al., 2021a).

Another Randomized, Double-Blind, Phase 3 Study was carried out in Japanese hemodialysis patients with anemia to compare the effects of DPD and darbepoetin. With most participants obtaining and maintaining hemoglobin levels within the target range, the results showed DPD’s non-inferiority to darbepoetin. Also, it was strongly advised to continuously check the hemoglobin response following the start of the DPD treatment. This study also suggested using less IV iron to regulate hemoglobin in individuals receiving DPD (Akizawa et al., 2020). Another recently published study revealed similar findings across several subgroups. The safety profile of DPD appeared to be largely comparable to that of darbepoetin alfa, and no unexpected safety issues were reported (Singh et al., 2022).

DPD has not yet been approved for patients with CKD anemia without dialysis, but ASCEND-ND (Anemia Studies in Chronic Kidney Disease: Erythropoiesis Via a Novel Prolyl Hydroxylase Inhibitor DPD-Non-Dialysis) trial has demonstrated safety and efficacy of DPD in treating anemia due to CKD in patients not undergoing dialysis. (Singh et al., 2021b). DPD would also be an excellent choice for patients preferring oral medication, improving compliance and accessibility. It would also suit the patients’ hyporesponsive to Erythropoiesis-stimulating agents (ESAs).

Data from the FDA’s adverse event reporting system (FAERS) may indicate an elevated risk of GI bleeding when HIF-PHIs are used to treat renal anaemia. DPD treatment may also raise the risk of vascular calcification in CKD patients with hyperphosphatemia. Serum copper excess may also be noted in some patients in the treatment.

Anemia in Heart Failure is a poor prognostic factor in both acute and chronic heart failure. Although anemia in heart failure is multifactorial, renal anaemia is one of the major causes. Coexisting renal and heart failure is reported to be associated with increased mortality (Al-Ahmad et al., 2001). The Study of Anemia in Heart Failure Trial (STAMINA-HeFT) (Ghali et al., 2008) and Reduction of Events With Darbepoetin Alfa in Heart Failure Trial (RED-HF Trial) (Swedberg et al., 2013) did not show significant clinical outcomes of using recombinant human erythropoietin (rhEP) in Heart failure patients. However, a randomized controlled study is approved to evaluate the safety and efficacy of DPD for anemia in patients with Heart failure and Chronic Kidney Disease. HIF-PH inhibition offers potential benefits for cardiac function independent of EPO production or iron metabolism regulation. In ischemic disease, hypoxia triggers the activation of HIF, initiating protective responses that safeguard ischemic tissues from hypoxic damage and enhance cardiovascular function by promoting re-oxygenation and repair. Moreover, research suggests that HIF inhibition could contribute to the transition from cardiac hypertrophy to heart failure (HF). Therefore, stabilizing HIF with these compounds could directly improve cardiac function and HF status (Iso et al., 2022). Depending upon the research results and future trials and analysis, DPD may be beneficial in treating renal anemia and improving cardiac function or reversing cardiac remodeling. This can open a new paradigm for treating Patients with Coexisting renal anemia and Heart Failure.

Provenance and peer review: Not Commissioned, externally peer-reviewed.

## Data Availability

The original contributions presented in the study are included in the article/Supplementary material, further inquiries can be directed to the corresponding author.
